# Spectrum analysis of inborn errors of metabolism for expanded newborn screening in a northwestern Chinese population

**DOI:** 10.1038/s41598-021-81897-y

**Published:** 2021-01-29

**Authors:** Ruixue Zhang, Rong Qiang, Chengrong Song, Xiaoping Ma, Yan Zhang, Fengxia Li, Rui Wang, Wenwen Yu, Mei Feng, Lihui Yang, Xiaobin Wang, Na Cai

**Affiliations:** 1 Center of Neonatal Disease Screening, Department of Clinical Genetics, Northwest Women’s and Children’s Hospital, 1616 Yanxiang Road, Xi’an, Shaanxi Province China; 2 Department of Pediatrics, Northwest Women’s and Children’s Hospital, 1616 Yanxiang Road, Xi’an, Shaanxi Province China; 3 Department of Child Healthcare, Northwest Women’s and Children’s Hospital, 1616 Yanxiang Road, Xi’an, Shaanxi Province China

**Keywords:** Clinical genetics, Mutation, Metabolomics, Metabolic disorders, Diagnosis, Disease prevention, Paediatrics, Public health

## Abstract

Expanded newborn screening facilitates early identification and intervention of patients with inborn errors of metabolism (IEMs), There is a lack of disease spectrum data for many areas in China. To determine the disease spectrum and genetic characteristics of IEMs in Xi'an city of Shaanxi province in northwest China, 146152 newborns were screening by MSMS from January 2014 to December 2019 and 61 patients were referred to genetic analysis by next generation sequencing (NGS) and validated by Sanger sequencing. Seventy-five newborns and two mothers were diagnosed with IEMs, with an overall incidence of 1:1898 (1:1949 without mothers). There were 35 newborns with amino acidemias (45.45%, 1:4176), 28 newborns with organic acidurias (36.36%, 1:5220), and 12 newborns and two mothers with FAO disorders (18.18%; 1:10439 or 1:12179 without mothers). Phenylketonuria and methylmalonic acidemia were the two most common disorders, accounting for 65.33% (49/75) of all confirmed newborn. Some hotspot mutations were observed for several IEMs, including *PAH* gene c.728G>A for phenylketonuria; *MMACHC* gene c.609G>A and c.567dupT, *MMUT* gene c.323G>A for methylmalonic acidemia and *SLC25A13* gene c.852_855del for citrin deficiency. Our study provides effective clinical guidance for the popularization and application of expanded newborn screening, genetic screening, and genetic counseling of IEMs in this region.

## Introduction

Inborn errors of metabolism (IEMs) are a phenotypically and genetically heterogeneous group of disorders caused by defective enzymes, cofactors, and transporters in metabolic pathways, leading to the accumulation of toxic substrates, the production of by-products, and reduced levels of products, which result in a series of clinical manifestations^1^. Since Garrod first proposed the concept of “inborn errors of metabolism” in 1909, more than 1000 different IEMs have been identified. While individual rare, the overall incidence has been shown to be upwards of 1 in 800^2^, IEMs can occur in any ethnic group and across every age. Clinical manifestations are complex and often non-specific, including acute metabolic crisis, epilepsy, metabolic acidosis, severe low blood sugar, high blood ammonia, and multiple organ damage. Some of them are amenable to treatment, if not treated timely, may cause irreversible physical disabilities, mental retardation, and even early death^3^.

Newborn screening (NBS) for IEMs can improve their prognosis and quality of life through early diagnosis and early intervention, recognizing as a huge success in field of public health of the last 50 years^4^. In the early 2000s, tandem mass spectrometry (MSMS) was introduced into many newborn screening programs, this technology allows inexpensive simultaneous detect dozens of amino acids and acylcarnitine profile in a single test, with commendable analytical accuracy and precision. Consequently, MS/MS has been applied wildly for the screening of IEMs that include most amino acid disorders, organic acidemias, and fatty acid oxidation defects ^1,5^.

In mainland China, MS/MS-based NBS was first piloted in Shanghai in 2003. There were 116000 newborns screened from 2003 to 2007, twenty IEMs were identified yielding an incidence of 1:5800^6^. However, subsequent studies have shown that the incidence, disease spectrum, and genetic characteristics of IEMs varied greatly in different regions of China, especially between the north and south. Furthermore, although MS/MS has been applied in China for many years, there are neither corresponding technical specifications in China nor expanded NBS data for many areas. Therefore, it is necessary to accumulate data on the clinical practice of MSMS and to study the IEMs disease spectrum and gene mutations in different regions to guide expanded NBS.

Expanded NBS program was begun in 2014 in Xi'an city, Shaanxi province, as an important economic and transportation hub city in northwest China. In this study, we present almost six years of experience with expanded NBS of 146152 newborns in the Northwest Women's and Children's Hospital, which is one of the largest tertiary hospitals for maternity and child care in northwest China, including the incidence, disease spectrum, and genetic characteristics of IEMs. These findings will lay the foundation for the establishment of expanded newborn screening systems with MSMS in this region.

## Results

### Performance of expanded newborn screening

Over 6 years, a total of 146152 newborns were screening by MSMS, consisting of 75672 males, 70463 females, and 17 infants with unknown sex. The rate of premature infants and low-birth-weight infants was 7.90% (11544/146152) and 6.63% (9683/146152), respectively. Initial screening was performed at an average age of 7.25 days at birth. The baseline characteristics of all the screened infants is shown in Table 1. After initial screening by MSMS, 3387 infants received positive results, yielding a positive rate of 2.32%. However, only 2729 infants (80.57%) were successfully recalled for retesting, and 305 cases still received positive. These infants were referred to specialists for differential diagnosis and genetic testing. In total, 75 infants and two mothers confirmed to have inborn errors of metabolism, with an overall incidence of 1:1898 via MS/MS newborn screening. The incidence of amino acid disorders, organic acid disorders, and fatty acid oxidation disorders was 1:4176, 1:5220, and 1:10439 (including two mothers), respectively. The overall positive predictive value (PPV) was 25.25%, and the overall false positive rate (FPR) was 0.16%. During the entire period reported, false-negative results were not observed, with overall sensitivity of 100% and specificity of 99.84%. The parameters of the study project are shown in Table 2.

**Table 1:** Baseline characteristics of newborns enrolled by expanded newborn screening program.

Category	Newborns without targeted IEMs	Confirmed IEM patients	*P*
**Number**	146077	75	
**Age at initial screening (days, mean ± SD)**	7.04 ± 5.63	7.63 ± 3.79	0.418
**Gender**			
Male	75633	39	0.995
Female	70427	36	
Unknown	17	0	
**Gestational age (weeks)**			
< 32	1054	0	< 0.001
32–36^+6^	10479	11	
≥ 37	134523	63	
Unknown	21	1	
**Birth weight (g)**			
< 2500	9677	6	0.049
2500–2999	24106	21	
≥ 3000	112266	48	
Unknown	28	0	
**Number of fetus**			
Singleton	145872	70	< 0.001
Twins	205	5	
Multiplets	0	0

**Table 2:** Parameters of the expanded newborn screening program.

Parameters	Value
Total screening number	146152
Positive cases of initial screening	3387
Positive rate of initial screening (%)	2.32
Positive recall cases of initial screening	2729
Positive recall rate of initial screening (%)	80.57
Positive cases after recall	305
Positive rate of recall (%)	11.18
Confirmed cases	77
Positive predictive value (%)	25.25
Sensitivity (%)	100.00
Specificity (%)	99.84
incidence	1:1898 (1:1949 without mother)
Amino acid disorders	1:4176
Organic acid disorders	1:5220
Fatty acid oxidation disorders	1:10439 (1:12179 without mother)

### Incidence and disease spectrum of IEMs

In this study, we identified a total of 14 types of IEMs, consisting of five amino acid disorders, five organic acidemias, and four fatty acid oxidation defects, which were confirmed in 35 newborns (45.45%), 28 newborns (36.36%), and 12 newborns and 2 mothers (18.18%), respectively. The highest incidence was phenylketonuria (PKU, 1:5220), accounting for 37.33% of confirmed newborns, the mean concentration of phenylalanine at initial screening was 1085.14 (Rang: 347.93–2766.42) μmol/L. Methylmalonic acidemia (MMA, 1:6960) was the next most common disorder, accounting for 28.00% of confirmed newborns, including 10 patients with combined MMA with homocysteinemia and 11 patients with isolated MMA. Citrin deficiency (CD, 1:36538) was a common amino acid disorder except PKU. Three infants each of 3-methyl-butanoyl CoA dehydrogenase deficiency (3MCCD), primary carnitine deficiency (PCD), short-chain acyl CoA dehydrogenase deficiency (SCADD), medium-chain acyl CoA dehydrogenase deficiency (MCADD), and very long-chain acyl CoA dehydrogenase deficiency (VLCADD) were identified, with an incidence of 1:48717. Other IEMs were very rare, with only 1 or 2 cases found in 146152 newborns. The disease spectrum and distribution of IEMs are shown in Table 3. In addition, we found two cases of maternal carnitine deficiency with free carnitine concentration of 2.2 μmol/L and 2.08 μmol/L, respectively. With the exception of PCD and ornithine aminotransferase deficiency (OTCD), the average value of the corresponding biomarkers with MSMS of all confirmed infants is higher than the upper limit of the reference value range, abnormal biomarkers were seen for details in Table 3.

**Table 3:** Abnormal MSMS markers and results statistics of all confirmed infants.

Disorders	*n*	Incidence	Abnormal MSMS markers	Concentration of initial screening (μmol/L) (mean, range)	Concentration of the second testing (μmol/L) (mean, range)	Reference range (μmol/L)
**Amino acid disorders**	**35**	**1:4176**				
Phenylketonuria (PKU)	28	1:5220	Phe	1085.14 (347.93–2766.42)	1840.29 (581.34–2369.21)	20.00–120.00
			Phe/Tyr	19.61 (4.74–50.53)	34.23 (5.75–41.70)	0.20–1.50
tyrosinemia type III	1	1:146152	Tyr	1177.00	904.71	25.00–280.00
			Tyr/Phe	23.62	26.09	0.60–5.00
Citrin deficiency (CD)	4	1:36538	Cit	139.70 (88.26–208.18)	315.55 (119.60–489.59)	6.50–37.00
			Cit/Phe	2.55 (1.19–4.02)	3.37 (2.73–4.16)	0.10–0.75
			Cit/Arg	4.87 (2.24–6.55)	5.16 (1.75–9.12)	0.25–4.50
Ornithine transcarbamylase deficiency (OTCD)	1	1:146152	Cit	3.3	6.14	6.50–37.00
			Cit/Phe	0.03	0.06	0.10–0.75
			Cit/Arg	0.10	0.06	0.25–4.50
Argininosuccinic aciduria (ASA)	1	1:146152	Cit	223.86	375.82	6.50–37.00
			Cit/Phe	3.73	11.37	0.10–0.75
			Cit/Arg	18.47	30.09	0.25–4.50
**Organic acid disorders**	**28**	**1:5220**				
Methylmalonic acidemia (MMA)	21	1:6960	C3	8.73 (1.69–29.28)	11.59 (2.34–67.68)	0.35–4.00
			C3/C2	0.85 (0.28–2.43)	1.28 (0.34–4.26)	0.03–0.18
			C3/Met	0.79 (0.14–2.84)	1.23 (0.18–4.04)	0.02–0.25
			C3/C0	0.78 (0.14–2.38)	0.65 (0.13–1.22)	0.02–0.22
3-Methylcrotonyl-CoA carboxylase deficiency (3MCCD)	3	1:48717	C4DC + C5OH	4.92 (3.05–7.23)	5.57 (4.28–7.30)	0.06–0.38
			(C4DC + C5OH)/C0	0.88 (0.15–1.27)	1.92 (0.51–3.29)	0–0.02
			(C4DC + C5OH)/C8	211.94 (50.83–361.5)	557.33 (428.00–514.00)	1.20–12.00
Glutaric acidemia I (GA-1)	2	1:73076	C5DC + C6OH	2.47 (1.95–2.99)	2.25 (1.66–2.84)	0.03–0.26
			(C5DC + C6OH)/C8	47.45 (65.00–29.90)	112.50 (83.00–142.00)	0.43–5.52
Holocarboxylase synthetas deficiency (HCS)	1	1:146152	C4DC + C5OH	0.90	1.35	0.06–0.38
			(C4DC + C5OH)/C0	0.03	0.04	0–0.02
			(C4DC + C5OH)/C8	22.50	45.00	1.20–12.00
Isobutyryl-CoA dehydrogenase deficiency (IBD)	1	1:146152	C4	2.50	1.36	0.09–0.45
			C4/C2	0.57	0.29	0–0.03
			C4/C3	1.51	1.72	0.06–0.35
**Fatty acid oxidation disorders**	**12**	**1:12179**				
Primary carnitine deficiency (PCD)	3	1:48717	C0	4.82 (2.31–8.07)	3.07 (1.94–4.28)	8.50–50.00
Short-chain acyl-CoA dehydrogenase deficiency (SCADD)	3	1:48717	C4	1.62 (1.39–1.89)	1.51 (0.95–2.23)	0.09–0.45
			C4/C2	0.10 (0.09–0.12)	0.17 (0.10–0.24)	0–0.03
			C4/C3	1.17 (0.97–1.46)	2.38 (2.16–2.56)	0.06–0.35
Medium chain acyl-CoA dehydrogenase deficiency (MCADD)	3	1:48717	C8	2.01 (1.60–2.26)	1.94 (0.67–3.70)	0.01–0.17
			C8/C2	0.21 (0.16–0.26)	0.29 (0.09–0.40)	0–0.10
			C8/C10	12.57 (8.69–14.55)	15.33 (5.58–24.33)	0.40–1.30
Very long chain acyl-CoA dehydrogenase deficiency (VLCADD)	3	1:48717	C14:1	3.97 (2.77–4.76)	2.68 (1.28–3.09)	0.02–0.19
			C14:1/C2	0.93 (0.69–1.34)	0.76 (0.60–1.03)	0–0.01
			C14:1/C16	0.95 (0.83–1.05)	0.79 (0.54–1.20)	0.01–0.11

### Genetic analysis in the confirmed cases

Except for PKU, all other confirmed infants accepted genetic testing by NGS, while only 14 (50.0%) of PKU infants received NGS testing because they could be diagnosed clearly and dietary therapy by specific biochemical markers. Their genetic testing results were shown in Table 4. Eighteen different mutations in the *PAH* gene were detected in fourteen patients with PKU, of which 77.8% (14/18) occurred in exon 7, and the most common mutation was c.728G>A (p. Arg243Gln) (6/28, 21.4%). Three patients with PKU were homozygous mutations in the *PAH* gene, none of them are inbreeding, and the others were compound heterozygous mutations.

**Table 4:** Gene mutation spectrum of 61 infants with IEMs.

Disorders	Gene (n)	Nucleotide variant	Amino acid variant	Mutation type	Region	Pathogenic	Allele frequency % (n)
**Amino acid disorders**							
Phenylketonuria (PKU)	*PAH* (14)	c.728G>A	p.Arg243Gln	Missense	E7	P	21.4 (6)
		c.721C>T	p.Arg241Cys	Missense	E7	P	10.7 (3)
		c.208_210del	p.Ser70del	In-frame	E3	P	7.1 (2)
		c.331C>T	p.Arg111Ter	Nonsense	E3	P	7.1 (2)
		c.611A>G	p.Tyr204Cys	Missense	E6	P	7.1 (2)
		c.137G>A	p.Gly46Asp	Missense	E2	LP	3.6 (1)
		c.442-1G>A		Splice site	I4	P	3.6 (1)
		c.482T>C	p.Phe161Ser	Missense	E5	P	3.6 (1)
		c.498C>G	p.Tyr166Ter	Nonsense	E5	P	3.6 (1)
		c.526C>T	p.Arg176Ter	Nonsense	E6	P	3.6 (1)
		c.754C>T	p.Arg252Trp	Missense	E7	P	3.6 (1)
		c.781C>T	p.Arg261Ter	Nonsense	E7	P	3.6 (1)
		c.782G>A	p.Arg261Gln	Missense	E7	P	3.6 (1)
		c.833C>T	p.Thr278Ile	Missense	E7	P	3.6 (1)
		c.842 + 2T>A		Splice site	I7	P	3.6 (1)
		c.842C>T	p.Pro281Leu	Missense	E7	P	3.6 (1)
		c.889C>T	p.Arg297Cys	Missense	E8	P	3.6 (1)
		c.1238G>C	p.Arg413Pro	Missense	E12	P	3.6 (1)
Tyrosinemia type III	*HPD* (1)	c.460G>A	p.Gly154Ser	Missense	E8	VUS	100.0 (1)
Citrin deficiency (CD)	*SLC25A13*(4)	c.852_855del	p.Met285fs	Frameshift	E9	P	50.0 (4)
		Not detected					50.0 (4)
Ornithine transcarbamylase deficiency (OTCD)	*OTC* (1)	c.958C>T	p.Arg320Ter	Nonsense	E9	P	100.0 (1)
Argininosuccinic aciduria (ASA)	*ASL* (1)	c.206A>G	p.Lys69Arg	Missense	E3	VUS	50.0 (1)
		c.637C>T	p.Arg213Ter	Nonsense	E9	P	50.0 (1)
**Organic acid disorders**							
Methylmalonic acidemia (MMA)	*MMACHC* (10)	c.609G>A	p.Trp203Ter	Nonsense	E4	P	20.0 (4)
		c.567dupT	p.Ile190fs	Frameshift	E4	P	20.0 (4)
		c.80A>G	p.Gln27Arg	Missense	E1	P	10.0 (2)
		c.217C>T	p.Arg73Ter	Nonsense	E2	P	5.0 (1)
		c.315C>G	p.Tyr105Ter	Nonsense	E3	P	5.0 (1)
		c.331C>T	p.Arg111Ter	Nonsense	E3	P	5.0 (1)
		c.394C>T	p.Arg132Ter	Nonsense	E3	P	5.0 (1)
		c.430-2A>C		Splice site	I4	P	5.0 (1)
		c.445_446del	p.Cys149fs	Frameshift	E4	P	5.0 (1)
		c.482G>A	p.Arg161Gln	Missense	E4	P	5.0 (1)
		c.648_650del	p.216_217del	In-frame	E4	LP	5.0 (1)
		c.656_658del	p.Lys220del	In-frame	E4	LP	5.0 (1)
		c.658_660del	p.Lys220del	In-frame	E4	P	5.0 (1)
	*MMUT* (11)	c.323G>A	p.Arg108His	Missense	E2	P	13.6 (3)
		c.729_730insTT	p.Asp244fs	Frameshift	E3	P	9.1 (2)
		c.1106G>A	p.Arg369His	Missense	E6	P	9.1 (2)
		c.1280G>A	p.Gly427Asp	Missense	E6	P	9.1 (2)
		c.1630_1631delGGinsTA	p.Gly544Ter	Nonsense	E9	P	9.1 (2)
		c.1630G>T	p.Gly544Ter	Nonsense	E9	P	9.1 (2)
		c.103C>T	p.Gln35Ter	Nonsense	E2	P	4.5 (1)
		c.599T>C	p.Ile200Thr	Missense	E3	LP	4.5 (1)
		c.682C>T	p.Arg228Ter	Nonsense	E3	P	4.5 (1)
		c.861C>G	p.Tyr287Ter	Nonsense	E4	P	4.5 (1)
		c.914T>C	p.Leu305Ser	Missense	E5	LP	4.5 (1)
		c.1038_1040del	p.346_347del	In-frame	E5	LP	4.5 (1)
		c.1676 + 11A>G		Splice site	I10	VUS	4.5 (1)
		c.1677-1G>A		Splice site	I10	P	4.5 (1)
		c.1741C>T	p.Arg581Ter	Nonsense	E10	P	4.5 (1)
3-Methylcrotonyl-CoA carboxylase deficiency (3MCCD)	*MCCC2* (3)	c.592C>T	p.Gln198Ter	Nonsense	E6	P	33.3 (2)
		c.659G>A	p.Gly220Glu	Missense	E7	P	16.7 (1)
		c.690_692del	p.230_231del	In-frame	E7	P	16.7 (1)
		c.1102delG	p.Gly368fs	Frameshift	E12	P	16.7 (1)
		c.1488G>C	p.Gln496His	Missense	E15	LP	16.7 (1)
Glutaric acidemia I (GA-1)	*GCDH* (2)	c.416C>G	p.Ser139Trp	Missense	E6	P	25.0 (1)
		c.533G>A	p.Gly178Glu	Missense	E7	P	25.0 (1)
		c.914C>T	p.Ser305Leu	Missense	E9	P	25.0 (1)
		c.1204C>T	p.Arg402Trp	Missense	E11	P	25.0 (1)
Holocarboxylase synthetas deficiency (HCS)	*HLCS* (1)	c.782delG	p.Gly261fs	Frameshift	E5	P	50.0 (1)
		c.947T>C	p.Ile316Thr	Missense	E5	VUS	50.0 (1)
Isobutyryl-CoA dehydrogenase deficiency (IBD)	*ACAD8* (1)	c.705 + 1G>A		Splice site	I6	P	50.0 (1)
		c.1129G>A	p.Gly377Ser	Missense	E10	P	50.0 (1)
**Fatty acid oxidation disorders**							
Primary carnitine deficiency (PCD)	*SLC22A5* (3)	c.1400C>G	p.Ser467Cys	Missense	E8	P	33.3 (2)
		c.51C>G	p.Phe17Leu	Missense	E1	P	16.7 (1)
		c.323_331del	p.108_111del	In-frame	E1	LP	16.7 (1)
		c.824 + 5G>C		Splice site	I4	VUS	16.7 (1)
		c.844C>T	p.Arg282Ter	Nonsense	E5	P	16.7 (1)
Short-chain acyl-CoA dehydrogenase deficiency (SCADD)	*ACADS* (3)	c.1031A>G	p.Glu344Gly	Missense	E9	P	33.3 (2)
		c.136C>T	p.Arg46Trp	Missense	E2	P	16.7 (1)
		c.164C>T	p.Pro55Leu	Missense	E2	P	16.7 (1)
		c.310_312del	p.Glu104del	In-frame	E3	LP	16.7 (1)
		c.950T>C	p.Met317Thr	Missense	E8	LP	16.7 (1)
Medium chain acyl-CoA dehydrogenase deficiency (MCADD)	*ACADM* (3)	c.985A>G	p.Lys329Glu	Missense	E11	P	66.7 (4)
		c.449_452del	p.Thr150fs	Frameshift	E6	P	16.7 (1)
		c.1238G>A	p.Arg413His	Missense	E12	VUS	16.7 (1)
Very long chain acyl-CoA dehydrogenase deficiency (VLCADD)	*ACADVL* (3)	c.1843C>T	p.Arg615Ter	Nonsense	E20	P	33.3 (2)
		c.298_299del	p.Gln100fs	Frameshift	E5	P	16.7 (1)
		c.996dupT	p.Ala333fs	Frameshift	E10	P	16.7 (1)
		c.1292A>G	p.Asp431Gly	Missense	E13	LP	16.7 (1)
		c.1396G>T	p.Asp466Tyr	Missense	E14	P	16.7 (1)

Genetic analysis of the 47 infants with non-PKU IEMs showed that 91.5% (43/47) had two variants in specific disease-causing genes. In total, sixty-four different mutations were detected in fourteen specific genes related to IEMs, including twenty-seven missense mutations, sixteen nonsense mutations, nine frameshift mutations, seven in-frame mutations, and five splice site mutations. Based on the American College of Medical Genetics and Genomics (ACMG) classification^7^, 75.0% (48/64) of variants were pathogenic, 15.6% (10/64) were likely pathogenic and 9.4% (6/64) were of uncertain significance. In addition, 76.6% (49/64) of variants were found in only one case. Compound heterozygous mutations were detected in 76.6% (36/47) of the cases, homozygous mutations were detected in 10.6% (5/47), none of them are inbreeding, mono-heterozygous mutations were detected in 10.6% (5/47), and hemizygous mutations were detected in 2.1% (1/47).

Among 21 patients diagnosed with MMA, 10 cases with combined MMA and homocysteinemia carried thirteen different mutations in the *MMACHC* gene mutations, the common mutations were c.609G>A (p. Trp203Ter) and c.567dupT (p. Ile190fs), accounting for 40% of *MMACHC* mutation alleles. While 11 cases with isolated MMA carried fifteen different mutations in the *MMUT* gene, the most common mutant allele was c.323G>A (p. Arg108His) (3/22, 13.6%), which involves the alteration of a conserved nucleotide located at the substrate binding (beta alpha) 8 barrel domain. This variant has been reported in multiple affected individuals mostly as compound heterozygotes and the patients' fibroblasts presented with a marked decrease in methylmalonyl-CoA mutase enzyme activity^8^. Heterozygous mutation of the *SLC25A13* gene (c.852_855del) was detected in 4 patients with CD, with a mutation frequency of 50%. In addition, some hotspot mutations were observed for other IEMs, including *MCCC2* gene c.592C>T, *SLC22A5* gene c.1400C>G, *ACADS* gene c.1031A>G, *ACADM* gene c.985A>G, and *ACADVL* gene c.1843C>T.

Among the confirmed patients, there were five cases of double pregnancy. Two cases, identical twins, carried compound heterozygous c.1630_1631delGGinsTA/c.323G>A mutations in the *MMUT* gene, were diagnosed with isolated MMA. Two other cases were assisted reproductive dizygotic twins, both of which were confirmed with MCADD, and a homozygous c. c.985A>G mutation in the *ACADM* gene was detected. Besides, one of the naturally conceived dizygotic twins had complex heterozygous c.136C>T/c.310_312delGAG mutation in the *ACADS* gene, was diagnosed as SCADD.

### Follow-up in the confirmed cases

Prior to diagnosis, most infants did not show typical clinical manifestations, while 14 patients developed suspicious symptoms, including poor response, milk refusal, infection, metabolic acidosis, hyperammonemia, hypoglycemia, etc. 11 patients died within 1–2 months of age due to infection, feed difficulties, acute metabolic crisis or multiple organ failure, including five patients with isolated MMA, three patients with VLCADD, two patients with GA-1 and one patient with OTCD. The details of these 14 patients are shown in Table 5. Exception for one child with argininosuccinic aciduria (ASA) whose parents refused to follow up, the remaining 63 children received regular follow-up, and all are developing normally at present. The longest follow-up period is approximately 6 years.

**Table 5:** Details of patients who became symptomatic before establishing the diagnosis.

No.	Diagnosis	Age of onset (days)	Symptom	Age at initial screening (days)	Age at confirmed diagnosis (days)	Age at start of treatment (days)	Prognosis
1	VLCADD	1	Poor response; milk refusal; infection; hypoglycemia	3	10	10	Die
2	GA-1	3	Poor response; milk refusal; encephalopathy; metabolic acidosis	8	14	14	Die
3	VLCADD	2	Poor response; hypoglycemia	7	10	10	Die
4	Isolated MMA	0.5	Poor response; milk refusal; encephalopathy; sepsis; metabolic acidosis; anemia	2	4	4	Die
5	GA-1	4	Poor response; milk refusal; encephalopathy; sepsis	4	6	6	Die
6	Isolated MMA	9	Poor response; milk refusal; metabolic acidosis; jaundice; congenital heart disease	9	11	11	Die
7	Isolated MMA	10	Poor response; milk refusal; metabolic acidosis; jaundice	11	11	11	Die
8	VLCADD	1	Hypoglycemia	4	9	10	Die
9	Isolated MMA	11	Poor response; milk refusal; metabolic acidosis; encephalopathy; infection; hypoglycemia; hyperammonemia	5	12	12	Under treatment
10	Isolated MMA	10	Poor response; milk refusal; anemia; encephalopathy; sepsis; seizures; hyperammonemia; congenital heart disease; coagulant function abnormality	10	11	11	Die
11	OTCD	2	Poor response; milk refusal; anemia; encephalopathy; sepsis; hyperammonemia; Congenital heart disease; coagulant function abnormality	3	4	4	Die
12	Isolated MMA	3	Poor response; milk refusal; congenital heart disease; pneumonia; metabolic acidosis; hypoglycemia; electrolyte disturbances	4	5	5	Under treatment
13	Isolated MMA	2	Poor response; milk refusal; sepsis; encephalopathy; metabolic acidosis; electrolyte disturbances; respiratory failure; hyperammonemia; coagulant function abnormality	3	6	6	Die
14	Isolated MMA	6	Poor response; milk refusal; anemia; encephalopathy; pneumonia; metabolic acidosis; hyperammonemia	5	8	8	Under treatment

## Discussion

There are no national detailed expanded NBS standards in China due to regional differences in the application of MSMS and lack of sufficient and valid studies. Some work has been done on the epidemiology, technical aspects and clinical validity of MS/MS screening in several provinces and cities in China, indicated that NGS combined with MS/MS is an enhanced plan for IEMs as well as disease spectrum and genetic characteristic of IEMs vary dramatically in different regions^9–13^. As far as we know this is the first single center report of the incidence, disease spectrum, and genetic characteristics of IEMs in a northwestern Chinese population.

A total of 146152 newborns underwent expanded NBS for IEMs until Dec 2019, and seventy-five infants and two mothers were diagnosed with IEMs in our 6-year study, and the average incidence of IEMs detected by MS/MS in Xi'an was 1:1898, which is higher than the average prevalence in previous reports from mainland China (1:3795)^14^ and the incidence in most Asian countries^15–18^. Moreover, compared with the incidence of IEMs in different regions in China, the local incidence was similar to that in Jining city (1:1941)^10^ in northern China and slightly higher than that in Quanzhou city (1:2804)^11^, Jiangsu province (1:2763)^9^, Zhejiang province (1:5626)^19^, Taiwan (1:6219)^20^, Hong Kong (1:4122)^21^ in south China. This indicates that IEMs are not rare in northwest China, However, facing the current situation that large-scale expanded NBS has not been carried out in this region, it is necessary to consider the incorporation of MSMS into the neonatal screening system.

Although there are significant differences in the disease spectrum of IEMs in different regions, it has been widely reported that PKU is the most common amino acid disorder. Moreover, numerous studies have shown that PKU is more common in northern China than in southern China, thus, our study further confirmed these conclusions. Patients with BH_4_ deficiency were not identified during the study period, which may further confirm the regional differences in PKU typing^12^. Genetic analysis showed that the most common mutation was c.728G>A in the *PAH* gene, which is in consistent with previous reports^22,23^.

Methylmalonic acidemia was the second most common IEM in this region and also had the highest incidence of organic acidemias (1:6960), which was slightly lower than reported in Jining city (1:5590)^10^ and Henan province (1:6032)^24^, but significantly higher than in Zhejiang province (1:46500)^25^, Jiangsu province (1:35734)^9^, Quanzhou city (1:121515)^11^, and Taiwan (1:101625)^20^ in southern China. Thus, our research provides further evidence that the incidence of MMA in northern China is higher than that in southern China. However, 21 patients were diagnosed with methylmalonic acidemia in this study, 52.4% of patients with isolated MMA and 47.6% with combined MMA with homocysteinemia, which were different from most reported conclusions that combined MMA with homocysteinemia caused by the *MMACHC* gene mutation is the common type of MMA in the mainland of China ^26,27^. It is worth noting that our study was performed at a single center, may result in selection bias. Therefore, it is necessary to continuously increase the samples from different cities in this region in order to obtain more objective and reliable information about the disease spectrum of MMA. c.609G>A and c.567dupT in the *MMACHC* gene were two common mutations, which is consistent with previous reports^26^. Similar to the report by Hu et al.^28^, in our population, c.323G>A was the most prevalent mutation in the *MMUT* gene. However, previous other studies have suggested that c.729_730insTT is the most common *MMUT* mutation in China^29,30^. This difference should be taken into account the small number of cases in this study and the impact of geographical differences.

Citrin deficiency was another common amino acid disorder detected in this study with an incidence of 1:36538, comparable with those reported in Jining city (1:39556)^10^ and Quanzhou city (1:36455)^11^. The incidence in our study was significantly higher than the calculated incidence based on the carrier rate of 1:940 in the *SLC25A13* gene in northern China^31^. However, a recent study conducted by Lin et al.^32^ on genetic screening for 29364 newborns at six NBS centers in southern, northern, and border regions of China, showed that the carrying rate of *SLC25A13* gene mutations were basically the same in these three regions (1/42, 1/43, and 1/56, respectively). Therefore, more data should be available to determine the true incidence and regional differences of CD. Nevertheless, a large number of studies have shown that c.852_855del in the *SLC25A13* gene is a hotspot mutation in Chinese patients with CD, with no regional differences in its incidence. The four patients diagnosed with CD in this study carried c.852_855del heterozygous mutations in the *SLC25A13* gene. But unfortunately, CD as an autosomal recessive disorder, we did not detect other mutations in these four patients, which may be related to the limited detection ability of target NGS technology to detect gene intron regions, regulatory regions, large fragment deletions / duplications and complicated structural rearrangements. In recent years, with the reduction in cost and widely application of NGS, newborn genetic screening has received more and more attention^33^. In terms of the above cases, biochemical screening is still irreplaceable. Of course, considering the previous reported cases of false negative results with MSMS screening, combining newborn metabolic and genetic screening may be a better choice.

In our study, fatty acid oxidation disorders accounted for only 18.18% of all confirmed cases, including PCD, SCADD, MCADD, and VLCADD, with 3 infants of each identified. However, if maternal carnitine deficiency was included in the analysis, it could be concluded that carnitine deficiency was more common in the region. c.1400C>G in the *SLC22A5* gene for PCD and c.1031A>G in the *ACADS* gene for SCADD were common mutations, consistent with previous reports^10–12,34^. However, of the 3 cases diagnosed with MCADD, 2 cases with dizygotic twins carried a homozygous c.985A>G (66.7%) mutation in the *ACADM* gene, so we think that the mutation frequency of c.985A>G seems to be overestimated in this study. c.985A>G is a known high-frequency mutation of MCADD in Caucasian populations, involved in approximately 90% of cases^35^, but it has not been found in Japan^36^, South Korea^37^ and Taiwan^20^.

In this study, there was one patient confirmed with HCS by expanded NBS. This disease is a type of multiple carboxylase deficiency (MCD) caused by abnormal biotin metabolism. The clinical manifestations are non-specific, and usually mainly include nervous system and skin damage. The results of MSMS showed elevated 3-hydroxypentanoylcarnitine. In mainland China, except for 3 cases of MCD confirmed in 1861262 newborns screened in Zhejiang province^25^, there were no patient reported in other regions by expanded NBS^9–11^. In addition, Han et al.^30^ screened 18303 patients with suspected IEMs by MSMS, 24 patients were diagnosed with HCS. Likewise, a comparative study of selective screening and expanded NBS in Asian countries suggested that larger number of patients with MCD were identified via selective screening, whereas very few were discovered with expanded NBS and it is easier to detect when dietary biotin insufficiency^38^. Therefore, the diagnosis of MCD depends more on the identification of clinicians, and of course, early diagnosis through expanded NBS can lead to effective intervention before the onset of clinical symptoms, thus reducing the risk of a poor prognosis.

This study had several limitations of note. With regard to MSMS screening efficiency, like most newborn screening centers in China, we did not use any second tier testing so that we had a high positive rate of initial screening. The Region4Stork (R4S) collaborative project has provided an optimized tool for laboratory management, clinical validation and interpretation of results of newborn screening by MS/MS, which effectively reduced the false positive rate and promoted the improvement of screening efficiency^39,40^. However, this system has not been widely used in China, and we still use traditional interpretation rules, which may also result in a high positive rate of initial screening. The artificial intelligence interpretation model based on localized big data under study is expected to further promote the interpretation of newborn screening results by MSMS. In addition, due to the lack of relevant screening policies and the inadequacy of education on expanded NBS, nearly 10% of the infants in this study were not recalled for secondary testing. Although we did not find false negative cases during the study period, which was related to the strict cut-off value we set, it also caused a high initial screening positive rate, which in turn caused a psychological and economic burden on neonatal guardians. The identification of false negative results mainly relies on child care management system. In this way, the current imperfect child care network may also lead to failure to effectively identify false negative cases. Therefore, how to further optimize the MSMS screening system and relevant policy support to improve screening efficiency will become the focus of our following work. Besides, a potential source of bias in sample acquisition may exit due to its single-center design, thus, our findings may not accurately reflect the prevalence of IEMs in Shaanxi province and even northwest China. However, considering the lack of massive information on expanded NBS in this region, so the incidence and disease spectrum of IEMs, including amino acid disorders, organic acidemia and fatty acid oxidation disorders derived from this study still have important reference value for the implementation of expanded NBS by MSMS in Northwest China.

## Conclusions

We presented expanded NBS results with MS/MS in a northwestern Chinese population. The prevalence, disease spectrum, and genetic characteristics of confirmed IEMs were initially clarified. Our experience showed that IEMs were not uncommon in northwest China, PKU and MMA were the most common IEMs in this region, which provided effective clinical guidance for the popularization and application of MS/MS with genetic analysis for NBS and diagnosis of IEMs. In addition, the mutational hotspots we found could be potential candidates for gene screening, this will be of value for genetic counseling and genetic diagnosis of IEMs. Of course, we also need to accumulate more clinical data to standardize and optimize expanded NBS with MSMS, and the combination of MS/MS with gene screening is necessary in future.

## Materials and methods

### Subjects and study protocol

From January 2014 to December 2019, A total of 146152 newborns were referred to expanded NBS by MS/MS at the Neonatal Screening Center of Northwest Women's and Children's Hospital in Xi'an, Shaanxi, China. The baseline characteristics of the enrolled infants is shown in Table 1. The study protocol was reviewed and approved by the Ethics Committee of Northwest Women's and Children's Hospital. Written informed contents were obtained from all the infants' parents.

All procedures were in accordance with the technical standards for newborn screening of the Ministry of Health of China (2010). In brief, blood samples were collected from the heel stick at more than 72 h after birth, spotted onto a Whatman 903 filter paper, air-dried at room temperature, and then sent to the laboratory for MSMS analysis. Blood specimens of preterm (< 32 weeks of gestational age) or low birth weight neonates (< 1000 g) were similarly assayed, but second specimens of these infants were collected and assayed again after one month of life. Newborns with positive initial screening results were recalled for repeated test, if the second test was still positive, the infant was transferred to a specialist for further diagnosis, treatment, and follow up. During the study period, the panel of screened disorders via MSMS was not officially regulated in China, and all disorders recommended in the US panel were screened for in our center.

### Expanded NBS by MSMS

Dried blood spot (DBS) samples were pre-processed following the instruction of the NeoBase non-derivative MSMS Kit (PerkinElmer, Waltham, United States). Each 3.2-mm diameter blood DBS sample was added to a PerkinElmer 96-well microtiter plate with 100 µL of extraction buffer containing amino acid and acylcarnitine internal standards. After incubation at 45 °C for 45 min with shaking in a PerkinElmer shaking incubator, 80 µL was transferred to a new microtiter plate and placed in an ACQUITY TQD mass spectrometer (Waters, Milford, MA, USA) for quantitative analysis^10,12^. The analytes included 11 amino acids and 31 acylcarnitine. the 11 amino acids were alanine (Ala), citrulline (Cit), arginine (Arg), glycine (Gly), leucine/isoleucine (Leu/lle/Pro-OH), methionine (Met), ornithine (Orn), phenylalanine (Phe), Valine (Val), Proline (Pro), and tyrosine (Tyr); the 31 acylcarnitine were C0, C2, C3, C3DC+C4OH, C4, C4DC+C5OH, C5, C5:1, C5DC+C6OH, C6, C6DC, C8, C8:1, C10, C10:1, C10:2, C12, C12:1, C14, C14:1, C14:2, C14OH, C16, C16:1, C16:1OH, C16OH, C18, C18:1, C18:1OH, C18:2, and C18OH.

The reference values were initially set by determining 3000 normal-term infants (0.5th–99.5th percentile), and then modified over time as more samples were analyzed and more clinical data were available to minimize both false positives and negatives. Each IEM had one or more indicators including metabolites and ratios, and the positive rules of IEMs are set by three experienced physicians (including two metabolic disease specialists and a laboratory technician). When MSMS results met the positive rules of IEMs, they were considered as positive. All the positive rules and disorder lists of IEMs were shown in Supplementary material.

### Genetic analysis

Genetic analysis was performed by amplicon target capture and next generation sequencing (NGS) technology (Uni-Medica, Shenzhen, China). Peripheral blood or DBS were collected from patients diagnosed with one kind of IEM by clinical and biochemical tests. Genomic DNA was extracted using Peripheral Blood or Dry Blood Spot Nucleic Acid Extraction Kits (Uni-Medica, Shenzhen, China) according to the manufacturer's protocol. The Qubit dsDNA HS assay (Invitrogen, Carlsbad, CA, USA) was used to quantify DNA concentrations. Then, the sequencing library was established using multiplex polymerase chain reaction (PCR) (Uni-Medica, Shenzhen, China) and quantified by Qubit and quantitative PCR. Library dilution and denaturation was performed following the kit instructions and sequenced on an Illumina HiSeq X10 (Illumina, San Diego, CA) platform. The data were analyzed using the Uni-Medical GTS Analysis System. Pathogenicity analysis of each variant was performed to comply with the ACMG guidelines^7^. Next, all variants identified by NGS were further validated by Sanger sequencing of the parents.

The panel contained 95 genes related to IEMs (*PAH*, *PTS*, *QDPR*, *FAH*, *TAT*, *HPD*, *BCKDHA*, *BCKDHB*, *DBT*, *CPS1*, *OTC*, *ASS1*, *NAGS*, *SLC25A13*, *ASL*, *ARG1*, *OAT*, *SLC25A15*, *CBS*, *MAT1A*, *PRODH*, *GLDC*, *MMACHC*, *MUT*, *MMAA*, *MMAB*, *PCCA*, *PCCB*, *IVD*, *GCDH*, *MCCC1*, *MCCC2*, *AUH*, *HMGCL*, *HLCS*, *BTD*, *ACAT1*, *ACADSB*, *ACAD8*, *HSD17B10*, *L2HGDH*, *SLC22A5*, *CPT1A*, *CPT2*, *SLC25A20*, *ACADS*, *ACADM*, *ACADVL*, *HADHA*, *HADHB*, *ETFA*, *ETFB*, *ETFDH*, *ETHE1*, *MLYCD*, *SMPD1*, *NPC1*, *NPC2*, *GBA*, *GLA*, *IDUA*, *IDS*, *GALNS*, *GLB1*, *ARSB*, *G6PC*, *SLC37A4*, *GAA*, *AGL*, *CYP11B1*, *GALC*, *PSAP*, *ARSA*, *ABCD1*, *ATP7B*, *GALT*, *ALDOB*, *DNAJC12*, *GCH1*, *PCBD1*, *MTHFR*, *DLD*, *PC*, *HADH*, *NADK2*, *SUCLA2*, *SUCLG1*, *LMBRD1*, *HCFC1*, *TAZ*, *GNMT*, *AHCY*, *MMADHC*, *MCEE*, and, *ABCD4)*.

### Diagnosis, treatment and follow-up

In consideration of early diagnosis and intervention for positive cases, we adopted a strategy of diagnosis and treatment at the same time. Diagnosis was made by combining clinical manifestations, individualized assistant examinations and genetic testing, of which genetic testing was a very important basis. For the positive cases after recall, individualized auxiliary examinations were conducted according to disease type, which included routine blood and urine tests, liver and kidney function assessment, myocardial zymogram, blood glucose, blood ammonia and blood gas analysis. For organic acid disorders and some cases of amino acid disorders, urine gas chromatography was used for differential diagnoses.

We conducted standard treatment and follow-up for all confirmed children according to the type of disease and clinical manifestation, which mainly included: (1) combination of diet and drug therapy, such as isolated MMA, glutaric aciduria type 1 (GA-1), etc.; (2) only diet treatment, such as PKU and CD, etc.; (3) only drug treatment, such as combined MMA with homocysteinemia, PCD and holocarboxylase synthetas deficiency (HCS), etc.; and (4) only regular follow-up, such as 3MCCD and SCADD, etc. these patients were followed up every 3 ~ 6 months, including blood MSMS, urine gas chromatography, blood biochemical analysis, imaging, physical examination and development assessment, etc. All infants with negative screening results were included in the children's health care management system for follow-up.

### Statistical analysis

Statistical analysis was performed using SPSS19.0. The difference of categorical data was compared using Chi-square test. The difference of measurement data was compared by t test between two groups. *P* < 0.05 was considered statistically significant.

### Ethical approval

The study protocol was reviewed and approved by the Ethics Committee of Northwest Women's and Children's Hospital.

### Informed consent

Informed consent was obtained from all families enrolled in the study.

## Supplementary Information

Supplementary Information.
