# Supplementary material for: Spectrum analysis of inborn errors of metabolism for expanded newborn screening in a northwestern Chinese population
Source: Sci Rep. 2021 Jan 29;11:2699. doi: 10.1038/s41598-021-81897-y (PMC7846761; doi:10.1038/s41598-021-81897-y)
Supplement: Supplementary file 1 — Supplementary Information. [file 41598_2021_81897_MOESM1_ESM.doc]

**Spectrum analysis of inborn errors of metabolism for expanded newborn screening in a northwestern Chinese population**

Ruixue Zhang ^a^, Rong Qiang^a*^, Chengrong Song^a^, Xiaoping Ma^a^, Yan Zhang^a^, Fengxia Li^b^, Rui Wang^a^, Wenwen Yu^a^, Mei Feng^b^, Lihui Yang^a^, Xiaobin Wang^a^, Na Cai^a^

*^a^ Center of Neonatal Disease Screening, Department of Clinical Genetics, Northwest Women*'*s and Children*'*s Hospital; 1616 Yanxiang Road, Xi'an, Shaanxi Province, China.*

*^b^ Department of Pediatrics, Northwest Women*'*s and Children*'*s Hospital; 1616 Yanxiang Road, Xi'an, Shaanxi Province, China.*

*^c^ Department of Child Healthcare, Northwest Women*'*s and Children*'*s Hospital; 1616 Yanxiang Road, Xi'an, Shaanxi Province, China.*

*Corresponding author: Rong Qiang, Email:* [*qiangrongshx@126.com*](mailto:qiangrongshx@126.com)

**Supplementary table 1 Disorders and their positive rules in expanded newborn screening panel**

| **Disorders** | **Abbreviation** | **Positive rule 1** | **Positive rule 2** | **Positive rule 3** |
| --- | --- | --- | --- | --- |
| Phenylketonuria; Benign hyperphenylalaninemia; BH4 deficiency | PKU; M-HPA; BH4 deficiency | PHE>100,PHE/TYR>1.5 | PHE>130 | PHE/TYR>2 |
| Citrin deficiency; Citrullinemia;Argininosuccinic acidemia | CD; CIT; ASA | CIT>40,ALA/CIT<8 | CIT>45 | CIT>35,CIT/PHE>0.75,ALA/CIT<9 |
| Hypermethioninemia | MET | MET>40,MET/PHE>0.8 | MET>65 |  |
| Maple syrup disease | MSUD | LEU+ILE+PRO-OH>300,LEU+ILE+PRO-OH/PHE>5.5,VAL>280 | LEU+ILE+PRO-OH>400 |  |
| Homocystinuria (attributable to cystathionine -synthase deficiency) | HCY | MET>35,MET/PHE>0.6,C3/MET<0.02 | MET<8,MET/PHE<0.1 |  |
| Tyrosinemia | TYR | TYR>350,PHE/TYR<0.3 | TYR>450 |  |
| Argininemia | ARG | ARG>50,ARG/PHE>1.0,ARG/ORN>0.35 | ARG>70 |  |
| Ornithine transcarbamylase deficiency; Carbamoylphosphate synthetase deficiency | OTC; CPS | CIT<6.5,CIT/PHE<0.15 | CIT<6 |  |
| Non-ketotic hyperglycinemia | NKH | GLY>1500 |  |  |
| Hyperornithinemia - hyperammonemia - homocitrullinuria syndrome | HHHS | ORN>450,ORN/CIT>25 |  |  |
| Hyperprolinemia | PRO | PRO>500,PRO/PHE>9.5 |  |  |
| Medium-chain acyl-CoA dehydrogenase deficiency | MCADD | C6>0.1,C8>0.15,C8/C2>0.01,C8/C10>1.3 | C8>0.3 |  |
| Very long-chain acyl-CoA dehydrogenase deficiency | VLCADD | C14:1>0.25,C14:1/C16>0.1,C14:1/C8>4 | C14:1>0.4 |  |
| Carnitine uptake defect | CUD | C0<9 |  |  |
| Long-chain L-3-hydroxyacyl-CoA dehydrogenase deficiency; Trifunctional protein deficiency | LCHAD; TFP | C16OH>0.05,C16OH/C16>0.03,C18:1OH>0.03,C18OH>0.02 |  |  |
| Short-chain acyl-CoA dehydrogenase deficiency; Isobutyryl-CoA dehydrogenase deficiency; Ethylmalonate encephalopathy | SCADD; IBG; EMA | C4>0.45,C4/C2>0.03 | C4>0.6 |  |
| Glutaric acidemia type II | GA-2 | C4>0.45,C8>0.17,C14:1>0.25 | C6>0.1,C12>0.28,C8/C2>0.01,C10>0.2,C14>0.3 |  |
| Carnitine palmitoyltransferase II deficiency; Carnitine/acylcarnitine translocase deficiency | CPT II; CACT | (C16+C18:1)/C2>0.36,C16>6,C0/(C16+C18)<2.5,C3/C16<0.35 | C16>6.3,C18:1>3 |  |
| Carnitine palmitoyltransferase I deficiency | CPT I | C0/(C16+C18)>55,C0>50 |  |  |
| Methylmalonic acidemia; Propionic acidemia | MMA; PA | C3/C2>0.2,C3>4 | C3>6 | C3/C2>0.25,MET<7,C3/MET>0.25 |
| Glutaric acidemia type I | GA-1 | C5DC+C6OH>0.24,(C5DC+C6OH)/C8>5 | C5DC+C6OH>0.3 |  |
| Isovaleric acidemia; 2-Methylbutyryl-CoA dehydrogenase deficiency | IVA, 2MBG | C5>0.5,C5/C3>0.4,C5/C2>0.03 | C5>0.6 |  |
| 3-Methylcrotonyl-CoA carboxylase deficiency; 3-Methylglutaconic aciduria; 3-Hydroxy-3-methyglutaric aciduria; Multiple carboxylase deficiency; 2-Methyl-3-hydroxybutyric aciduria; | 3-MCC; 3MGA; HMG;MCD; 2M3HBA | C4DC+C5OH>0.8 | C4DC+C5OH>0.5,(C4DC+C5OH)/C8>10 |  |
| β-Ketothiolase deficiency | BKT | C4DC+C5OH>0.38,C5:1>0.02,C3DC+C4OH>0.3 |  |  |
| Malonic acidemia | MAL | C3DC+C4OH>0.3,(C3DC+C4OH)/C10>3.5，(C3DC+C4OH)/C8>6 | C3DC+C4OH>0.5 |  |
